# Legal Notes for Institutional Workers

**Published:** 1912-05-04

**Authors:** 


### LEGAL NOTES FOR INSTITUTIONAL WORKERS.

(The Editor will be pleased to answer Legal queries in this column.)

### Consent to Post-mortem Examinations.

Anyone who is in a responsible position at a hospital is probably well aware of the legal conditions under which a post-mortem examination may be made; but from time to time events show that this knowledge cannot be taken for granted and so a brief summary may be useful. Sometimes, of course, a post-mortem is made as the result of a coroner's order; otherwise it can only take place under severe restrictions. The law is to be found in the Anatomy Act, 1832, which provides that any executor "or other party having lawful possession of the body of any deceased person" may permit the body to undergo anatomical examination, unless such person have during his life expressed a desire to the contrary, or unless "the surviving husband, or wife, or any known relative of the deceased shall require the body to be interred without such examination." It will be noticed that "the executor or other party" is under no obligation to communicate with the relatives; but it is believed to be the general practice at hospitals not to make any examination until every reasonable effort has been made to communicate with the persons likely to object. The "other party having lawful possession" is not defined by statute. If there is no executor it would seem that the husband, or wife, or nearest relative is entitled to possession if any one of these persons is going to bear the expenses of interment. Failing any relative, the person in whose house the deceased has died is probably entitled to possession; and if this be correct hospital authorities are entitled to the possession of the body of any person dying in the hospital whose relations cannot be found. It has been held, at any rate, that in this case the duty of burial falls upon the hospital.

### The Loss of the "Titanic"—Some Possible Medico-Legal Questions.

The appalling sacrifice of life which followed upon the loss of the ill-fated "Titanic" is not unlikely to bring a number of cases before the courts which are of interest both to the medical profession and to lawyers. In the first place there will probably be countless claims under the Workmen's Compensation Act. Although the lawyer can imagine certain lines of defence which might avail the employers, they are not likely to be seriously disputed. But questions of survivorship may arise. Where property is settled by will, or by a deed *inter vivos*, its devolution often depends upon the question whether one of two parties died first. In the case of husband and wife, both being drowned at sea, there is a legal presumption that a man will be able to maintain the cruel struggle for existence longer than a woman; but in the case of two men—*e.g.* father and son, or two brothers, the courts have been unable to lay down any definite rule. Finally, there will have to be numerous applications to the Probate Court for leave to presume the death of many of those unfortunate passengers who left Southampton on the "Titanic" but failed to reach New York.

### The Causation of Consumption.

The wording of the various Compensation Acts has led to many decisions which to the medical profession have seemed most extraordinary; both ordinary common sense and the accepted doctrines of medicine and surgery have many times been ignored in verdicts which have proved to be sound in law. In one recent case, however, the line of argument proved to be too far-fetched, and the claim failed. A widow applied for compensation in the Salford County Court for the death of her husband from consumption. The claim was based upon the fact that some time previously he suffered an injury to the left eye while at work, which resulted in loss of the sight in that eye. This was supposed to have preyed upon his mind to such an extent that consumption followed. A medical witness for the applicant with some hardihood supported this view, which was opposed by two other witnesses, one of them a well-known consultant. The Judge said that he thought it possible that the fatal disease was either caused or accelerated by the accident, but that the connection could not be held to be proved to the "reasonable satisfaction" of the Court. In order to succeed, such a claim must seem so probable that no other hypothesis could be accepted.

### Libels on Hospital Staffs.

"Another hospital scandal" is an all-too-frequent announcement on the placards which are exposed in public places to promote the sale of the less reputable papers. The "scandal" is probably founded upon the *obiter dictum* of some coroner, or the evidence of a disgruntled person at a police court; but if it gives a pretext for a headline it is thought good enough. We can hear the news editor say to himself, "At any rate, a hospital cannot bring suit for libel, so there is no risk to be run." Institutions have become so much accustomed to these forms of attack that they can generally afford to ignore them. A conspiracy of silence is most effective; but it should be remembered that an attack on an institution may involve a libel upon some person or persons connected with it, and he or they can seek the protection of the courts. The Lord Chancellor said in a recent famous libel case: The question for the jury is not "Who was meant," but "Who is hit." So if it was alleged that "the house surgeon at the X hospital turned A. B. out to die in the street," it is apprehended that any one of the house surgeons at the X hospital could bring suit for libel if he could call witnesses to show that they knew or thought that he was the person referred to. *Id certum est quod certum reddi potest!*

